# Job Scheduling in Cloud Computing Using a Modified Harris Hawks Optimization and Simulated Annealing Algorithm

**DOI:** 10.1155/2020/3504642

**Published:** 2020-03-11

**Authors:** Ibrahim Attiya, Mohamed Abd Elaziz, Shengwu Xiong

**Affiliations:** ^1^School of Computer Science and Technology, Wuhan University of Technology, Wuhan, China; ^2^Department of Mathematics, Faculty of Science, Zagazig University, Zagazig, Egypt; ^3^School of Computer Science and Technology, Huazhong University of Science and Technology, Wuhan, China

## Abstract

In recent years, cloud computing technology has attracted extensive attention from both academia and industry. The popularity of cloud computing was originated from its ability to deliver global IT services such as core infrastructure, platforms, and applications to cloud customers over the web. Furthermore, it promises on-demand services with new forms of the pricing package. However, cloud job scheduling is still NP-complete and became more complicated due to some factors such as resource dynamicity and on-demand consumer application requirements. To fill this gap, this paper presents a modified Harris hawks optimization (HHO) algorithm based on the simulated annealing (SA) for scheduling jobs in the cloud environment. In the proposed HHOSA approach, SA is employed as a local search algorithm to improve the rate of convergence and quality of solution generated by the standard HHO algorithm. The performance of the HHOSA method is compared with that of state-of-the-art job scheduling algorithms, by having them all implemented on the CloudSim toolkit. Both standard and synthetic workloads are employed to analyze the performance of the proposed HHOSA algorithm. The obtained results demonstrate that HHOSA can achieve significant reductions in makespan of the job scheduling problem as compared to the standard HHO and other existing scheduling algorithms. Moreover, it converges faster when the search space becomes larger which makes it appropriate for large-scale scheduling problems.

## 1. Introduction

Scientific computing is a promising field of study that is usually associated with large-scale computer modeling and simulation and most often requires a massive amount of computing resources [1]. For instance, scientific applications in various domains such as computational materials science, high energy physics, molecular modeling, earth sciences, and environmental computing involve the production of massive datasets from simulations or large-scale experiments. Hence, analyzing and disseminating these datasets among researchers/scientists located over a wide geographic area requires high power of computing that goes beyond the capabilities of a single machine. Therefore, given the ever-growing data produced by scientific applications and the complexities of the applications themselves, it becomes prohibitively slow to deploy and execute such applications on traditional computing paradigms.

To cope with the complexities and ever-increasing computational demand of those large-scale scientific applications, the concept of cloud computing is introduced. It provides elastic and flexible resources of computing (e.g., CPU, storage, memory, and networks) which can be rapidly provisioned and released with minimal management effort or service provider interaction [2]. These cloud services can be automatically as well as easily scaled up or down and delivered to the end customers based on a pay-per-use payment model. The major services offered by cloud providers can be classified as infrastructure as a service (IaaS), platform as a service (PaaS), and software as a service (SaaS). At the bottom of the cloud computing stack is the IaaS model. In this model, fundamental resources of the computing such as CPU, storage, memory, and bandwidth are offered. The next layer in the stack is PaaS. PaaS provides a high-level integrated environment to build, test, deploy, and host developer-created or -acquired applications. The SaaS is allocated at the top of the stack, and it is a software delivery model in which applications or services are provided over the Internet so that end users can access them through a web browser. This model allows users to utilize online software instead of the locally installed one. SaaS has now become a popular delivery model for service apps such as web mail, Google Docs, and social networking applications.

There is a consensus in the researcher community and IT industries that the growth in cloud computing will far exceed that of other computing paradigms [3]. This is due to the following reasons: First, the cloud offers a practical solution to solve the issue of resource lack and greatly reduces the expenses of purchasing and maintaining the physical resources [4, 5]. Second, it provides virtually infinite resources at different prices to properly serve different application requirements, in a dynamic and elastic way. As a result, the emphasis of computing has recently been pushed onto cloud platforms. On the contrary, with the growing adoption of cloud services (especially the IaaS model) by several institutions, there has been a huge amount of computing tasks that are implemented in the cloud computing (CC) environment. Since the computing resources are heterogeneous and geographically distributed, there are compromises in terms of communication cost, system integration, and system performance. Thus, in order to efficiently execute the cloud user requests and utilize the distributed resources in an appropriate way, a scheduling policy needs to be in place.

Indeed, many algorithms have been introduced to tackle the job scheduling problem in a CC environment. In the early stage, various heuristics were developed to address job scheduling problems which produce optimal schedule solutions for small-sized problems [6]. However, the quality of solutions generated by these heuristic strategies deteriorates severely as the problem scale and the number of objectives to be optimized increase. In addition, the solutions produced by these heuristic techniques depend largely on certain underlining rules, and they are generated at high operating cost [7]. In contrast, metaheuristic techniques have proven to be effective, robust, and efficient in solving a wide variety of real-world complex optimization problems. This can be attributed to their ability to employ a set of candidate solutions for the purpose of traversing the solution space, rather than using a single candidate solution as with heuristic techniques. This feature of metaheuristic algorithms makes them outperform other optimization methods. Some of the most popular metaheuristic techniques that have been introduced to solve the cloud job scheduling problem include genetic algorithm (GA) [8], particle swarm optimization (PSO) [9], ant colony optimization (ACO) [10], tabu search [11], BAT algorithm [12], simulated annealing (SA) algorithm [13], symbiotic organisms search (SOS) [14], and cuckoo search (CS) algorithm [15]. Although some of these algorithms have shown promising improvements in finding the global optimal solution for the job scheduling problem in the cloud, they are all suffering from premature convergence and difficulty to overcome local minima especially when faced with a large solution space [16]. These limitations often lead to suboptimal job scheduling solutions which affect the system performance and also violate quality of service (QoS) guarantees. This indicates that there is an urgent need for new adaptive and efficient algorithms to find the global optimal solution for the cloud job scheduling problem.

Recently, Heidari et al. [17] proposed a population-based, nature-inspired optimization technique called the Harris hawks optimizer (HHO), which mimics the social behavior of Harris' hawks in nature. It is mainly inspired by the cooperative behavior and chasing strategy of Harris hawks. The exploration and exploitation phases of the HHO are modeled by exploring a prey, performing a surprise pounce, and then attacking the intended prey. The Harris hawks optimizer can demonstrate a variety of attacking strategies according to the dynamic nature of circumstances and escaping behaviors of the prey. Local optima avoidance and smooth transition from exploration to exploitation are among the major advantages of the HHO algorithm. According to these behaviors, the HHO has been applied to several global optimization and real-world engineering problems, including image segmentation [18, 19], feature selection [20], spatial assessment of landslide susceptibility [21], parameter estimation of photovoltaic cells [22], image denoising [23], and others [24–29]. However, the HHO suffers from some limitations that affect its performance such that its exploration ability is weaker than its exploitation ability, and this leads to degradation of the performance of convergence and the quality of the solution. In addition, there is plenty of room to investigate the potential improvements in terms of speed of convergence and quality of solutions generated by the HHO.

A promising research direction, which hybridizes one or more heuristics/metaheuristics to leverage the strengths of these algorithms while reducing their impediments, has attracted tremendous attention from researchers in various research domains. In this study, an integrated version of the HHO algorithm with simulated annealing (HHOSA) was proposed as an attempt to improve the rate of convergence and local search of HHO. To the best of our knowledge, no research study found in the literature tries to investigate the above-mentioned improvements with the performance of HHO while tackling the job scheduling problem. Hence, the main motivation of our study is to propose the hybrid HHOSA approach for optimization of job scheduling in cloud computing.

The major contributions of this paper can be summarized as follows:

1. Design and implementation of a hybrid version of HHO and SA for optimum job scheduling in cloud computing
2. Empirical analysis of the convergence trend for 15 different job scheduling instances using HHOSA and other evaluated algorithms
3. Performance comparison of HHOSA against the original HHO and other popular metaheuristics in terms of makespan and performance improvement rate (%)

The remainder of this paper is structured as follows: Section 2 presents a review of related works on existing job scheduling algorithms.  Section 3 formulates the job scheduling problem and presents the original HHO and SA algorithms. Design of the proposed HHOSA algorithm and its description are introduced in Section 4. Performance evaluation and discussion of the achieved results are provided in Section 5. Finally, conclusions and outlines for potential future works are given in Section 6.

## 2. Related Works

Recently, there has been a significant amount of interest in using metaheuristics (MHs) for solving different problems in several domains. The MH methods have many advantages such as flexibility and simplicity. Therefore, they can used to solve a different number of optimization problems that cannot be solved by traditional methods. In addition, they are easy to implement. According to these advantages, several studies established that the MH methods provide good results for the task scheduling problems in cloud computing than other traditional methods [16, 30]. The authors in [31, 32] provided a comprehensive review of various metaheuristics that have been developed for solving the task scheduling problem in cloud computing.

Guo et al. [33] presented a task scheduling approach depending on a modified PSO algorithm which aims to minimize the processing cost of user tasks through embedding crossover and mutation operators within the PSO procedure. The results showed that the modified PSO provides a good performance especially with a large-scale dataset. Similarly, Khalili and Babamir [34] developed a modified version of PSO by using different techniques to update its inertia weight. Then, this version was applied in a cloud environment to reduce the makespan of the workload scheduling. Alla et al. [35] provided two hybrid PSO versions which depend on dynamic dispatch queues (TSDQ) for task scheduling. The first approach combined the fuzzy logic with PSO (TSDQ-FLPSO), while the second approach combined simulated annealing with PSO (TSDQ-SAPSO). The results of TSDQ-FLPSO outperform those of the other methods including the TSDQ-SAPSO. Other works related to the application of PSO to task scheduling in cloud computing have been reported in the literature [36–38].

Genetic algorithm (GA) has also been applied to solve the task scheduling problem. For example, Rekha and Dakshayini [39] introduced the GA to find a suitable solution for the task allocation which seeks to reduce task completion time. The results of the GA are assessed by comparing it with the simple allocation methods in terms of throughput and makespan. The results illustrated that the GA outperforms other compared algorithms. The authors in [40] presented a modified version of the GA using a heuristic-based HEFT to find a suitable solution for the static task scheduling in the cloud. Akbari et al. [41] developed new operators to enhance the quality of the GA and used this model to enhance the results of task scheduling. In [42], the authors provided a modified GA called MGGS, which is a combination of the GA and greedy strategy. The MGGS is compared with other approaches, and the experimental results established its ability to find a suitable solution for the task scheduling problem. Besides, a modified version of the GA is proposed in [43] which combines the GA with PSO. The developed method, called GA-PSO, is provided to solve the task scheduling so as to minimize the makespan and cost. Additionally, a hybrid genetic-particle swarm optimization (HGPSO) method [44] is proposed to solve the task scheduling issue. In the HGPSO, the user tasks are forwarded to a queue manager, and then the priority is computed and proper resources are allocated. This algorithm focuses on optimizing parameters of QoS. Moreover, there are several hybrid techniques in which the PSO and GA are combined and utilized to handle the task scheduling in cloud computing [45–47]. Other works combining the GA and fuzzy theory have been proposed in [48]. The proposed approach, called FUGE, aims to perform proper cloud task scheduling considering the execution cost and time. The performance of FUGE is compared with that of other task scheduling approaches, and the results illustrate its efficiency using different measures such as execution time, the average degree of imbalance, and execution cost.

Moreover, the ant colony optimization (ACO) has become one of the most popular task scheduling methods [49–53]. Keshk et al. [54] proposed a task scheduling algorithm based on the ACO technique in the cloud computing environment. The modified version, called MACOLB, aims at minimizing the makespan time while balancing the system load. Also, the firefly algorithm (FA) has been applied to enhance the results of the job scheduling such as in [55]. The FA is proposed as a local search method to improve the imperialist competitive algorithm (ICA), and this leads to enhancing the makespan. Esa and Yousif [56] proposed the FA to minimize the execution time of jobs, compared the results with those of the first-come first-served (FCFS) algorithm, and found the FA outperforms the FCFS algorithm. For more details about using the FA, refer [57–59]. In addition, the salp swarm algorithm was proposed to improve the placement of the virtual machine in cloud computing as in [60]. Braun et al. [61] introduced a comparison between eleven algorithms for mapping/assigning/scheduling independent tasks onto heterogeneous distributed computing systems. These methods include opportunistic load balancing (OLB), minimum execution time (MET), minimum completion time (MCT), min-min, max-min, duplex, SA, GA, tabu, and A*∗* heuristic. In addition, the operators of SA are combined with the operators of the GA, and this version is called genetic simulated annealing (GSA). In this algorithm (i.e., GSA), the mutation and crossover are similar to the GA, while the selection process is performed by using the cooling and temperature of SA.

Furthermore, the symbiotic organisms search (SOS) has attracted much attention recently as an alternative approach to solve task scheduling. In [62], a discrete version of the SOS (DSOS) is proposed to find the optimal scheduling of tasks in cloud computing. The comparison results illustrated that the DSOS is more competitive and performs better than both SAPSO and PSO. Moreover, it converges faster in the case of large instances. The work in [63] presents a modified version of the SOS based on the SA procedure to solve the task scheduling issue in cloud computing. The developed model, called SASOS, is compared with the original SOS, and the results showed the high performance of the SASOS in terms of makespan, convergence rate, and the degree of imbalance. Abdullahi et al. [6] proposed a multiobjective large-scale task scheduling technique based on improving the SOS using chaotic maps and chaotic local search (CLS) strategy. The aim of using the chaotic map is to construct the initial solutions and replace the random sequence to improve the diversity. In contrast, the CLS strategy is used to update the Pareto fronts. The proposed CMOS algorithm produced significant results when compared with other methods, including ECMSMOO [64], EMS-C [65], and BOGA [66]. These algorithms aim to achieve balancing between the makespan and the cost without any computational overhead. The experimental results demonstrate that the CMSOS has potentials to enhance the QoS delivery.

To sum up, according to the previous studies, the above-mentioned MH methods provide high ability to find a suitable solution for job scheduling in cloud computing. However, this performance still needs much improvement with a focus on finding suitable operators to balance between the exploration and the exploitation.

## 3. Background

### 3.1. Model and Problem Formulation

The IaaS cloud is a very common model from the perspective of resource management; thus, scheduling in such systems has gained great attention especially from the research community [67]. The IaaS cloud model provides computing resources as virtual resources that are made accessible to consumers through the Internet [68, 69]. Indeed, virtualization is one of the primary enablers for cloud computing. With virtualization technology, all physical resources in the cloud environment are represented as virtual machines (VMs) [70]. Hence, cloud providers must supply their customers with infinite virtualized resources in accordance with the service level agreement (SLA) [71] and must decide the best resource configuration to be utilized according to their profitability principles.

In our problem description, there is a general framework that focuses on the interaction between the cloud information service (CIS), the cloud broker, and the VMs [72]. When user requests are submitted to the cloud, these requests are forwarded to the cloud broker who maintains its characteristics and resource requirements. The cloud broker will then consult the CIS to determine the services required to process the received requests from the consumer and then map the job requests on the detected services. For the sake of clarity, suppose there is a set of independent jobs *J*={*J*_1_, *J*_2_,…, *J*_*n*_} that are submitted by the cloud consumers to be processed. The processing requirements of a job are referred to as job length and are measured in million instructions (MI). The cloud broker is then responsible for assigning those jobs onto the set of VMs available within the cloud data center to meet the users' demands. Let VM={VM_1_, VM_2_,…, VM_*m*_} denote the set of VMs. Each VM_*j*_ is a set of computing entities with limited capabilities (e.g., CPU power, memory space, storage capacity, and network bandwidth) [73]. It is assumed that VMs are heterogeneous and their CPU capabilities (measured in MIPS (millions of instructions per second)) are used to estimate the execution time of user requests. This indicates that a job executed on different VMs will encounter different execution cost. Our aim is to schedule a set of submitted jobs on available VMs to achieve higher utilization of resources with minimal makespan. We formulate our scheduling problem based on the “expected time to compute” (ETC) model [74]. The ETC is defined as the expected execution time of all jobs to compute on each VM obtained by using the ETC matrix as in equation (1). This means that based on the specifications of the VMs and submitted jobs, the cloud broker computes an *n* × *m* ETC matrix, where *n* is the total number of user jobs and *m* is the total number of available VMs. The element ETC_*i*,*j*_ represents the expected time for VM_*j*_ to process the job *J*_*i*_.

(1) $$\begin{array}{c} {\text{ETC}}_{i,j}=\left[\begin{array}{cccc} {\text{ETC}}_{1,1} & {\text{ETC}}_{1,2} & \ldots & {\text{ETC}}_{1,m}\\ {\text{ETC}}_{2,1} & {\text{ETC}}_{2,2} & \ldots & {\text{ETC}}_{2,m}\\ \ldots & \ldots & \ldots & \ldots \\ {\text{ETC}}_{n,1} & {\text{ETC}}_{n,2} & \ldots & {\text{ETC}}_{n,m} \end{array}\right], \end{array}$$

(2) $$\begin{array}{c} {\text{ETC}}_{i,j}=\frac{\text{Job}.{\text{Length}}_{i}}{\text{VM}.{\text{Power}}_{j}}, \end{array}$$

where ETC_*i*,*j*_ refers to the expected execution time of the *i*^th^ job on the *j*^th^ VM, Job.Length_*i*_ is the length of the job *i* in terms of MI, and VM.Power_*j*_ is the computing capacity of VM_*j*_ in terms of MIPS.

The main purpose of job scheduling is to find an optimal mapping of jobs to resources that optimize one or more objectives. In addition, the most common objective noticed in the reviewed literature is the minimization of job completion times, also known as makespan [75]. Thus, the aim of this study is to reduce the makespan of job scheduling, by finding the best allocation of virtual resources to jobs on the IaaS cloud.

For any schedule *X*, the makespan (MKS) is the maximum completion time and is calculated as follows:

(3) $$\begin{array}{c} \text{MKS}\left(X\right)=\underset{j\in 1,2,\ldots ,m}{\max}\sum_{i=1}^{n}{\text{ETC}}_{i,j}, \end{array}$$

where *m* and *n* are the number of machines and jobs, respectively. ETC_*i*,*j*_ is defined in equation (2). Then, the scheduling problem is formulated mathematically as

(4) $$\begin{array}{c} \text{Obj}:f\left(X\right)=\min\text{MKS}\left(X\right)=\min\underset{j\in 1,2,\ldots ,m}{\max}\sum_{i=1}^{n}{\text{ETC}}_{i,j}. \end{array}$$

### 3.2. Simulated Annealing Algorithm

The simulated annealing (SA) algorithm is considered one of the most popular single-solution-based optimization algorithms that emulate the process of annealing in metallurgy [76, 77]. The SA begins by setting an initial value for a random solution *X* and determining another solution *Y* from its neighborhood. The next step in SA is to compute the fitness value for *X* and *Y* and set *X*=*Y* if *F*(*Y*) ≤ *F*(*X*).

However, SA has the ability to replace the solution *X* by *Y* even when the fitness of *Y* is not better than the fitness of *X*. This depends on the probability (Prob) that is defined in the following equation:

(5) $$\begin{array}{c} \text{Prob}=e^{-\Delta E/kT},\\ \Delta E=F\left(X\right)-F\left(Y\right), \end{array}$$

where *k* and *T* are the Boltzmann constant and the value for the temperature, respectively. If Prob > rand, then *X*=*Y*; otherwise, *X* will not change. The next step is to update the value of the temperature *T* as defined in the following equation:

(6) $$\begin{array}{c} T=\beta \times T, \end{array}$$

where *β* ∈ [0,1] represents a random value. The final steps of the SA algorithm are given in Algorithm 1.

### 3.3. Harris Hawks Optimizer

**Figure 1:**
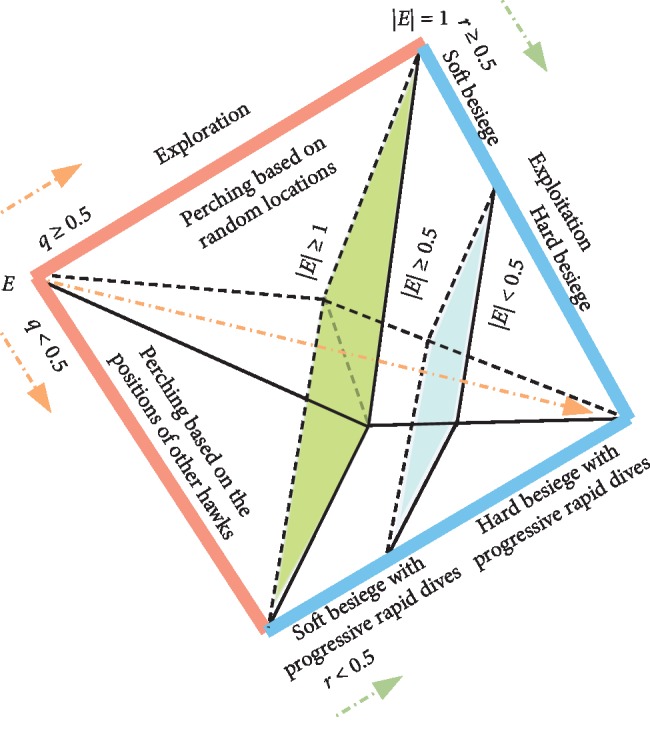
Stages of the HHO [17].

The Harris hawks optimizer (HHO) is a new metaheuristic algorithm developed to solve global optimization problems [17]. In general, the HHO simulates the behaviors of the hawks in nature during the process of searching and catching their prey. Similar to other MH methods, the HHO performs the search process during two stages (i.e., exploration and exploitation) based on different strategies, as given in Figure 1. These stages will be explained in more detail in the following sections.

#### 3.3.1. Exploration Phase

At this stage, the HHO has the ability to update the position of the current hawk (*X*_*i*_, *i*=1,2,…, *N*) (where *N* indicates the total number of hawks) depending on either a random position of another hawk (*X*_*r*_) or the average of the positions (*X*_Avg_) for all hawks. The selection process has the same probability to switch between the two processes, and this is formulated as in the following equation:

(7) $$\begin{array}{c} X_{i}\left(t+1\right)=\left\{\begin{array}{cc} X_{r}\left(t\right)-r_{1}\left|X_{r}\left(t\right)-2r_{2}X\left(t\right)\right|, & q\geq 0.5,\\ \left(X_{b}\left(t\right)-X_{Avg}\left(t\right)\right)-\omega , & q<0.5, \end{array}\right. \end{array}$$

where *ω*=*r*_3_(LB+*r*_4_(UB − LB)) and *X*_Avg_ is formulated as

(8) $$\begin{array}{c} X_{\text{Avg}}\left(t\right)=\frac{1}{N}\sum_{i=1}^{N}X_{i}\left(t\right). \end{array}$$

In general, the main goal of this stage is to broadly distribute the hawks across the search space. In the following section, we will discuss how hawks change their status from exploration to exploitation.

#### 3.3.2. Changing from Exploration to Exploitation

In this stage, the hawks transfer to exploitation based on their energies *E* which are formulated as

(9) $$\begin{array}{c} E=2E_{0}\left(1-\frac{t}{t_{\max}}\right), \end{array}$$

where *E*_0_ ∈ [−1,1] represents a random value and *t*_max_ and *t* represent the total number of iterations and the current iteration.

#### 3.3.3. Exploitation Phase

The exploitation stage of the HHO is formulated using several strategies and a few random parameters used to switch between these strategies [17]. These strategies are formularized as follows: (1) soft besiege, (2) hard besiege, (3) soft besiege with progressive rapid dives, and (4) hard besiege with progressive rapid dives.

- Soft besiege: in this phase, the hawks move around the best one, and this is formulated by using the following equations:

(10) $$\begin{array}{c} X\left(t+1\right)=\Delta X\left(t\right)-E\left|J\times X_{b}\left(t\right)-X\left(t\right)\right|, \end{array}$$

(11) $$\begin{array}{c} \Delta X\left(t\right)=X_{b}\left(t\right)-X\left(t\right). \end{array}$$

(ii) Hard besiege: in this phase, the hawks update their position based on the distance between them and the best hawk as given in the following equation:

(12) $$\begin{array}{c} X\left(t+1\right)=X_{b}\left(t\right)-E\times \left|\Delta X\left(t\right)\right|. \end{array}$$

(iii) Soft besiege with progressive rapid dives: at this stage, it is supposed that the hawks have the ability to choose the following actions. This can be captured from the following equation:

(13) $$\begin{array}{c} Y=X_{b}\left(t\right)-E\left|J\times X_{b}\left(t\right)-X\left(t\right)\right|. \end{array}$$

  The Levy flight (LF) operator is used to calculate the rapid dives during this stage, and this is formulated as

(14) $$\begin{array}{c} Z=Y+S\times \text{LF}\left(D\right), \end{array}$$

  where *S* represents a random vector with size 1 × *D* and *D* is the dimension of the given problem. In addition, the LF operator is defined as

(15) $$\begin{array}{c} \text{LF}\left(x\right)=0.01\times \frac{u\times \sigma }{\left|v{|}^{1/\beta }\right.},\\ \sigma ={\left(\frac{\Gamma \left(1+\beta \right)\times \sin\left(\pi \beta /2\right)}{\Gamma \left(\left(1+\beta \right)/2\right)\times \beta \times 2^{\left(\left(\beta -1\right)/2\right)}}\right)}^{1/\beta }, \end{array}$$

  where *u* and *v* represent random parameters of the LF operator and *β*=1.5.
  The HHO aims to select the best from *Y* and *Z* as defined in equations (13) and (14), and this is formulated as

(16) $$\begin{array}{c} X\left(t+1\right)=\left\{\begin{array}{cc} Y, & \mathrm{if}\;F\left(Y\right)<F\left(X\left(t\right)\right),\\ Z, & \mathrm{if}\;F\left(Z\right)<F\left(X\left(t\right)\right). \end{array}\right. \end{array}$$

(iv) Hard besiege with progressive rapid dives: in this stage, the hawks finish the exploitation phase with a *hard besiege*, and this is performed using the following equation:

(17) $$\begin{array}{c} X\left(t+1\right)=\left\{\begin{array}{cc} Y^{\prime }, & \mathrm{if}\;F\left(Y^{\prime }\right)<F\left(X\left(t\right)\right),\\ Z^{\prime }, & \mathrm{if}\;F\left(Z^{\prime }\right)<F\left(X\left(t\right)\right), \end{array}\right. \end{array}$$

where *Z*′ and *Y*′ are computed as in the following equations:

(18) $$\begin{array}{c} Y^{\prime }=X_{b}\left(t\right)-E\left|J\times X_{b}\left(t\right)-X_{\text{Avg}}\left(t\right)\right|,\\ Z^{\prime }=Y^{\prime }+S\times \text{LF}\left(D\right). \end{array}$$

For clarity, the previous strategies are performed depending on the energy of the hawks *E* and a random number *r*. For example, considering |*E*|=1.5 means that the operators of the exploration phase are used to update the position of the hawk. When *E*=0.7 and *r*=0.6, the soft besiege strategy will be used. In case of *E*=0.3 and *r*=0.6, the hawks' position will be updated using the operators of the hard besiege strategy. In contrast, when *E*=0.7 and *r*=0.3, the hawks' position will be updated using the soft besiege with progressive rapid dives strategy. Otherwise, the hard besiege with progressive rapid dives strategy will be used to update the current hawk.

The final steps of the HHO algorithm are illustrated in Algorithm 2.

## 4. Proposed Algorithm

In this section, an alternative approach for job scheduling in cloud computing is developed which depends on the modified HHO using the operators of SA. The main objective of using SA is to employ its operators as local operators to improve the performance of the HHO. The steps of the developed method are given in Algorithm 3.

In general, the developed HHOSA method starts by determining its parameters in terms of the number of individuals *N* in the population *X*, the number of jobs *n*, the number of virtual machines *m*, and the total number of iterations *t*_max_. The next step is to generate random solutions *X* with the dimension *N* × *n*. Each solution *x*_*i*_ ∈ *X* has *n* values belonging to the interval [1, *m*]. Thereafter, the quality of each solution is assessed by computing the fitness value (*F*) that is defined in equation (4). Then, *x*_*b*_ is determined. Finally, the individuals' set *X* will be updated according to the operators of the HHOSA method. The process of updating *X* is iterated until the terminal criteria are reached. A description with more details for each step of the proposed approach will be illustrated in the following sections.

### 4.1. Initial Stage

At this stage, a set of random integer solutions is generated which represents a solution for the job scheduling. This process focuses on identifying the dimension of the solutions that is given by the number of jobs *n*, as well as the lower lb and upper ub boundaries of the search space, which are determined in our job scheduling model by 1 and *m*, respectively. Therefore, the process of generating *x*_*i*_ ∈ *X*(*i*=1,2,…, *N*) is given by the following equation:

(19) $$\begin{array}{c} x_{ij}=\text{Rod}\left({\text{lb}}_{ij}+\text{rnd}\times \left({\text{ub}}_{ij}-{\text{lb}}_{ij}\right)\right),\;j=1,2,\ldots ,n, \end{array}$$

where each value of *x*_*i*_ belongs to an integer value in the interval [1, *m*] (i.e., *x*_ij_ ∈ [1, *m*]). Meanwhile, the Rod function is applied to round the value to the nearest whole number. rnd represents a random number belonging to [0, 1].

For more clarity, consider there are eight jobs and four machines, and the generated values for the current solution are given in *x*_*i*_ as $x_{i}=\left[\begin{array}{cccccccc} 4 & 1 & 4 & 4 & 2 & 3 & 1 & 3 \end{array}\right]$. In this representation, the first value in *x*_*i*_ is 4, and this indicates that the first job will be allocated on the fourth machine. Thus, it can be said that the first, third, and fourth jobs are allocated on the fourth machine, while the second and seventh jobs will be allocated on the first machine. Meanwhile, the sixth and eighth jobs will be allocated on the third machine, whereas the second machine will only execute the fifth job.

### 4.2. Updating Stage

This stage begins by computing the fitness value for each solution and determining *x*_*b*_ which has the best fitness value *F*_*b*_ until the current iteration *t*. Then, the operators of either SA or HHO will be used to update the current solution, and this depends on the probability (Pr_*i*_) of the current solution *x*_*i*_ that is computed as

(20) $$\begin{array}{c} {\mathrm{Pr}}_{i}=\frac{F_{i}}{\sum_{i=1}^{N}F_{i}}. \end{array}$$

The operators of the HHO will be used when the value of Pr_*i*_ > *r*_pr_; otherwise, the operators of SA will be used. Since the value of *r*_pr_ has a larger effect on the updating process, we made it automatically adjusted as in the following equation:

(21) $$\begin{array}{c} r_{\text{pr}}=L_{{\mathrm{Pr}}_{i}}+\text{rand}\times \left(U_{{\mathrm{Pr}}_{i}}-L_{{\mathrm{Pr}}_{i}}\right), \end{array}$$

where *L*_Pr_*i*__ and *U*_Pr_*i*__ are the minimum and maximum probability values for the *i*-th solution, respectively. When the HHO is used, the energy of escaping *E* will be updated using equation (9). According to the value of *E*, the HHO will go through the exploration phase (when |*E*| > 1) or exploitation phase (when |*E*| < 1). The value of *x*_*i*_ will be updated using equation (7) in the case of the exploration phase. Otherwise, *x*_*i*_ will be updated using one strategy from those applied in the exploitation phase which are represented by equations (10)–(17). The selection strategy is based on the value of the random number *r* and the value of |*E*| (which assumes its value may be in the interval [0.5, 1] or less than 0.5). Meanwhile, if the current solution is updated using the SA (i.e., Pr_*i*_ ≤ *r*_pr_), then a new neighboring solution *Y* to *x*_*i*_ will be generated and its fitness value *F*_*Y*_ will be computed. In the case of *F*_*Y*_ < *F*_*x*_*i*__, then *x*_*i*_=*Y*; otherwise, the difference between *F*_*x*_*i*__ and *F*_*Y*_ is computed (i.e., *δ*=*F*(*x*_*i*_) − *F*(*Y*)) and the value of Prob will be checked (as defined in equation (5)). If its value is less than *r*_5_ ∈ [0,1], then *x*_*i*_=*Y*; otherwise, the value of the current solution will not change.

The next step after updating all the solutions using either HHO or SA is to check the termination conditions; if they are reached, then running the HHOSA is stopped and the best solution is returned; otherwise, the updating stage is repeated again.

## 5. Experimental Results and Analysis

In this section, we present and discuss various experimental tests in order to assess the performance of our developed method. In Section 5.1, we introduce a detailed description of the simulation environment and datasets employed in our experiments.  Section 5.2 explains the metrics used for evaluating the performance of our HHOSA algorithm and other scheduling algorithms in the experiments. Finally, Section 5.3 summarizes the results achieved and provides some concluding remarks.

### 5.1. Experimental Environment and Datasets

This section describes the experimental environment, datasets, and experimental parameters. To evaluate the effectiveness of the developed HHOSA approach, the performance evaluations and comparison with other scheduling algorithms were performed on the CloudSim simulator. The CloudSim toolkit [78] is a high-performance open-source framework for modeling and simulation of the CC environment. It provides support for modeling of cloud system components such as data centers, hosts, virtual machines (VMs), cloud service brokers, and resource provisioning strategies. The experiments were conducted on a desktop computer with Intel Core i5-2430M CPU @ 2.40 GHz with 4 GB RAM running Ubuntu 14.04 and using CloudSim toolkit 3.0.3.  Table 1 presents the configuration details for the employed simulation environment. All the experiments are performed by using 25 VMs, hosted on 2 host machines within a data center. The processing capacity of VMs is considered in terms of MIPS.

For experiments, both synthetic workload and standard workload traces are utilized for evaluating the effectiveness of the proposed HHO technique. The synthetic workload is generated using a uniform distribution, which exhibits an equal amount of small-, medium-, and large-sized jobs. We have considered that each job submitted to the cloud system may need different processing time, and its processing requirement is also measured in MI.  Table 2 summarizes the synthetic workload used.

Besides the synthetic workload, the standard parallel workloads that consist of NASA Ames iPSC/860 and HPC2N (High-Performance Computing Center North) are used for performance evaluation. NASA Ames iPSC/860 and HPC2N set log are among the most well-known and widely used benchmarks for performance evaluation in distributed systems. Jobs are supposed to be independent, and they are not preemptive. More information about the logs used in our experiments is shown in Table 3.

For the purpose of comparison, each experiment was performed 30 times. The specific parameter settings of the selected metaheuristic (MH) methods are presented in Table 4.

### 5.2. Evaluation Metrics

The following metrics are employed to evaluate the performance of the HHOSA method developed in this paper against other job scheduling techniques in the literature.

#### 5.2.1. Makespan

It is one of the most commonly used criteria for measuring scheduling efficiency in cloud computing. It can be defined as the finishing time of the latest completed job. Smaller makespan values demonstrate that the cloud broker is mapping jobs to the appropriate VMs. Makespan can be defined according to equation (3).

#### 5.2.2. Performance Improvement Rate (PIR)

It is utilized to measure the percentage of the improvement in the performance of each method with regard to other compared methods as presented in equation (22). This provides an insight into the performance of the presented HHOSA against the state-of-the-art approaches in the literature. The PIR is defined as follows:

(22) $$\begin{array}{c} \text{PIR}\left(\%\right)=\frac{\left(S-S^{\prime }\right)}{S^{\prime }}*100, \end{array}$$

where *S*′ and *S* are the fitness values obtained by the proposed algorithm and the compared one from the related literature, respectively.

### 5.3. Result Analysis and Discussion

This section introduces the result analysis and discussion of experimentation of the proposed HHOSA job scheduling strategy. To objectively evaluate the performance of the HHOSA strategy, we have validated it over five well-known metaheuristic algorithms, namely, particle swarm optimization (PSO) [79], salp swarm algorithm (SSA) [80], moth-flame optimization (MFO) [81], firefly algorithm (FA) [82], and Harris hawks optimizer (HHO) [17].

**Figure 2:**
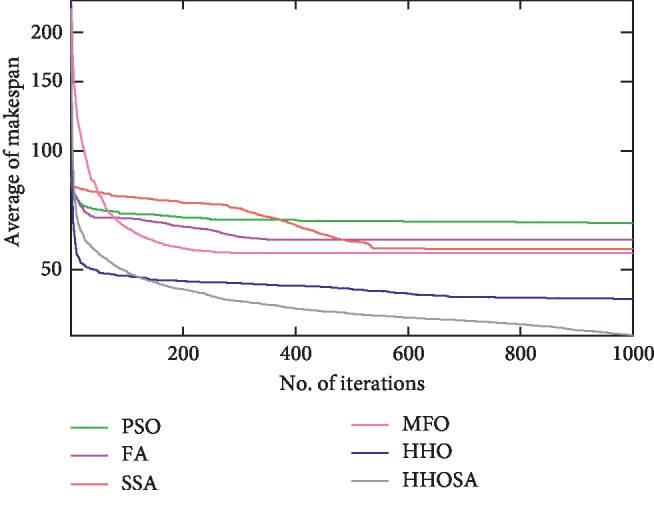
Convergence trend for synthetic workload (200 jobs).

**Figure 3:**
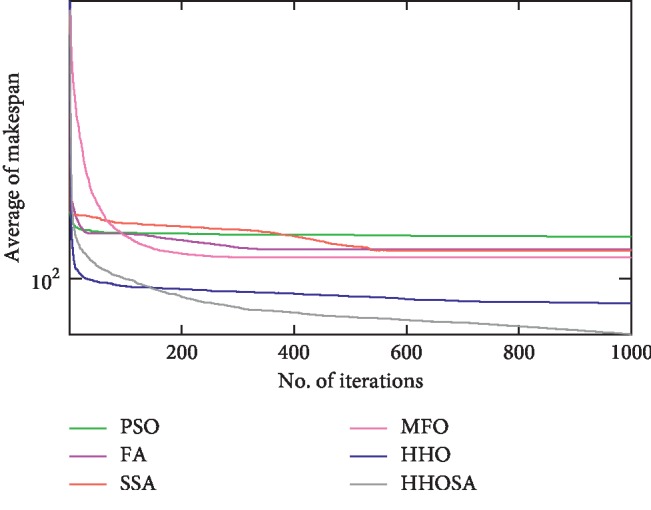
Convergence trend for synthetic workload (400 jobs).

**Figure 4:**
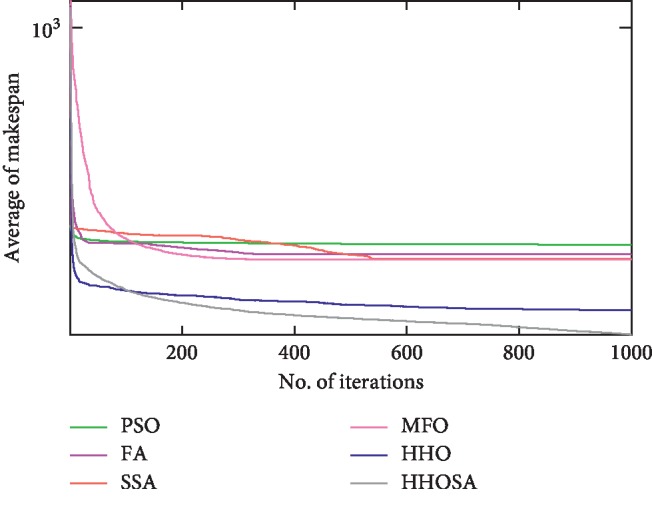
Convergence trend for synthetic workload (600 jobs).

**Figure 5:**
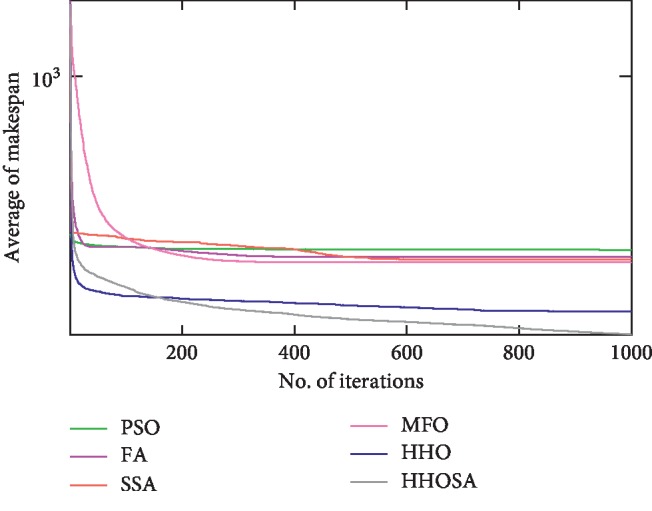
Convergence trend for synthetic workload (800 jobs).

**Figure 6:**
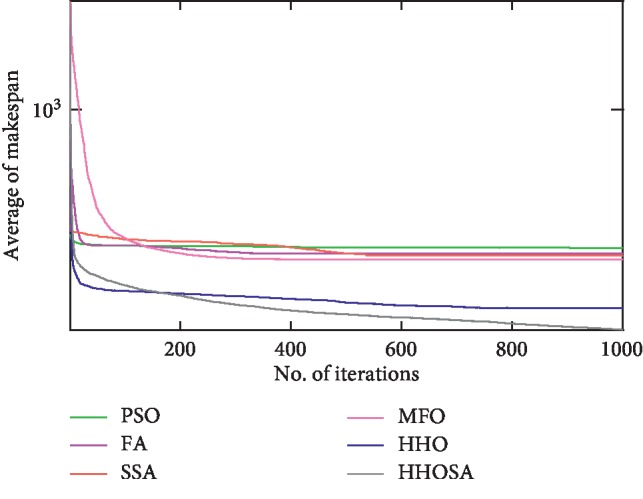
Convergence trend for synthetic workload (1000 jobs).

**Figure 7:**
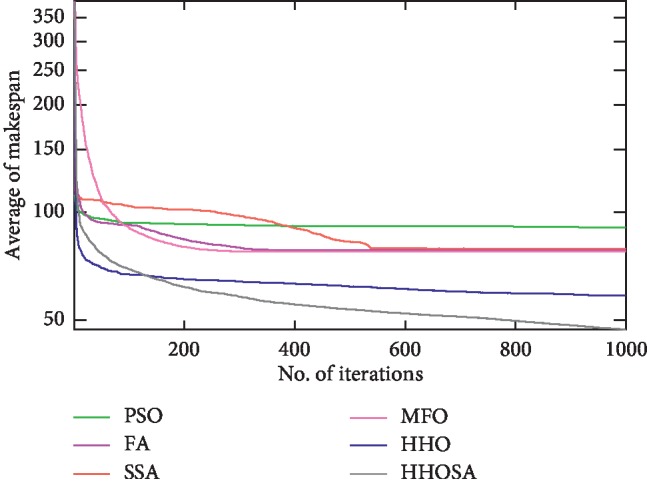
Convergence trend for real workload NASA iPSC (500 jobs).

**Figure 8:**
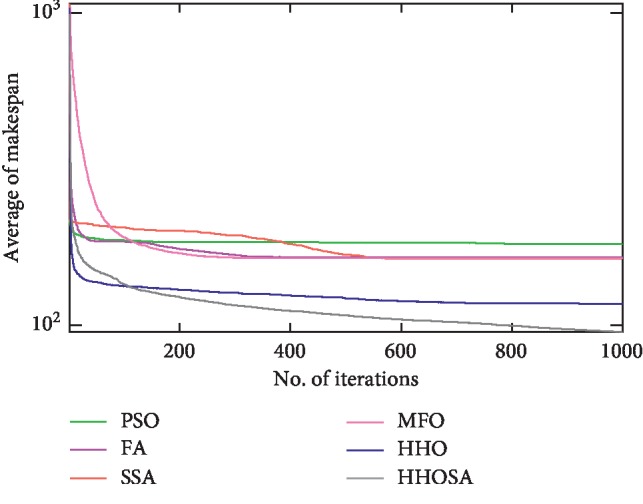
Convergence trend for real workload NASA iPSC (1000 jobs).

**Figure 9:**
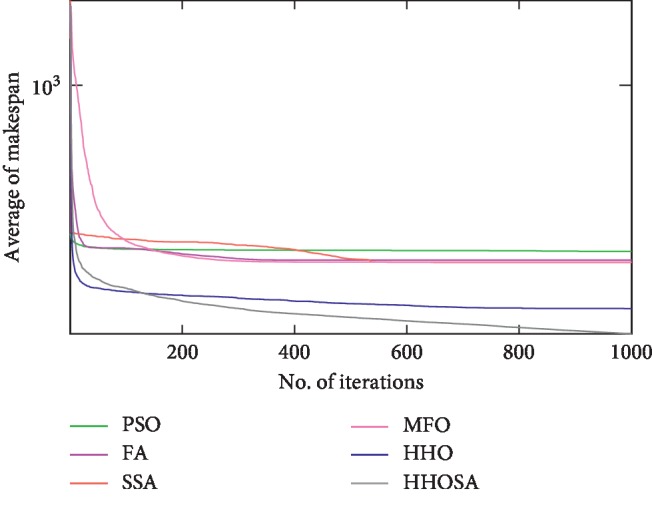
Convergence trend for real workload NASA iPSC (1500 jobs).

**Figure 10:**
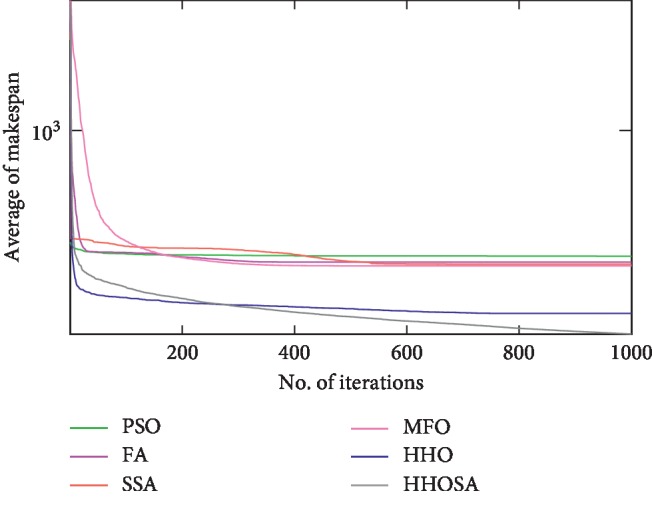
Convergence trend for real workload NASA iPSC (2000 jobs).

**Figure 11:**
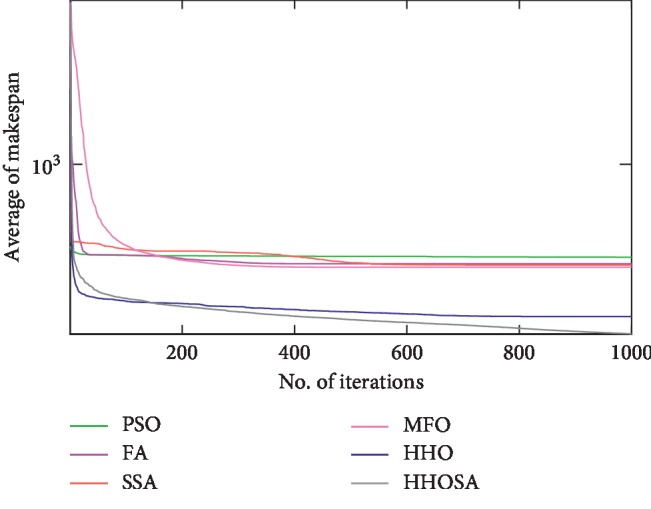
Convergence trend for real workload NASA iPSC (2500 jobs).

**Figure 12:**
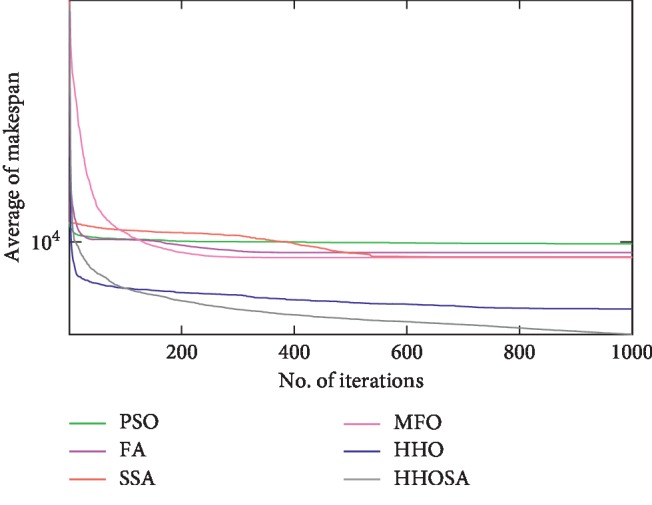
Convergence trend for real workload HPC2N (500 jobs).

**Figure 13:**
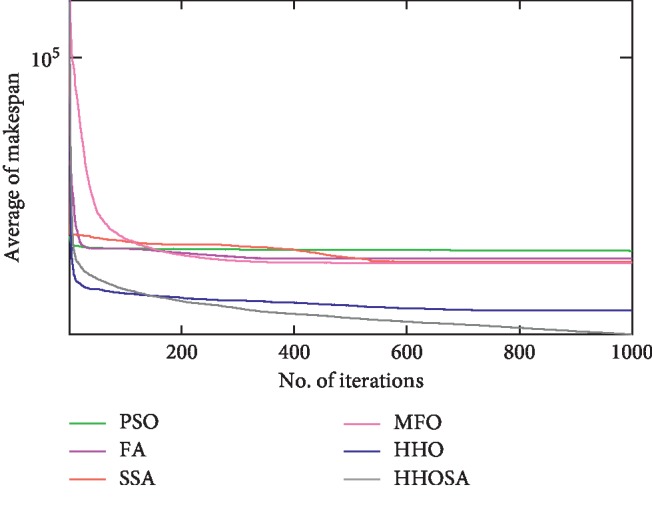
Convergence trend for real workload HPC2N (1000 jobs).

**Figure 14:**
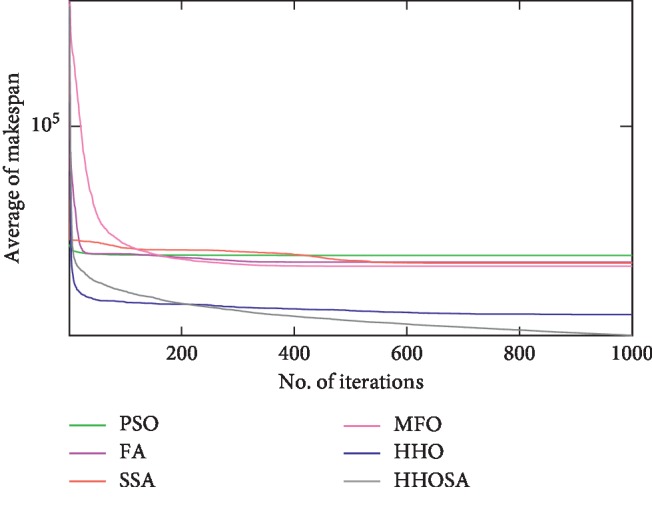
Convergence trend for real workload HPC2N (1500 jobs).

**Figure 15:**
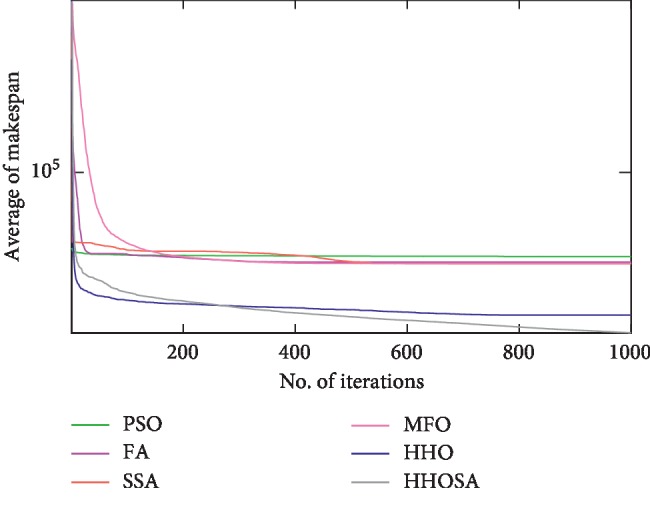
Convergence trend for real workload HPC2N (2000 jobs).

**Figure 16:**
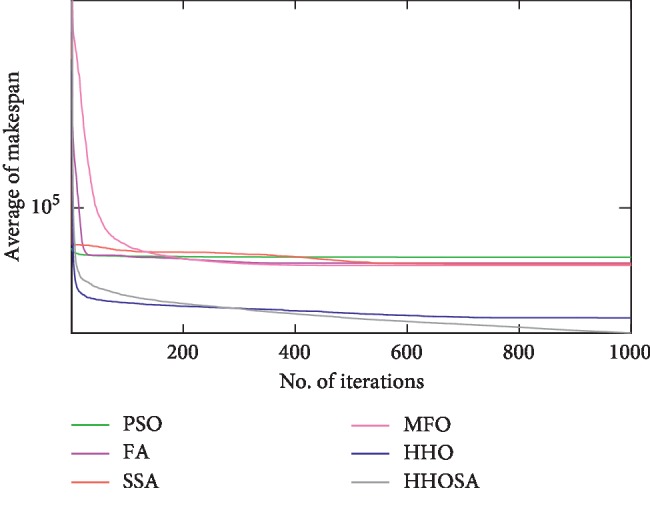
Convergence trend for real workload HPC2N (2500 jobs).

To display the performance of HHOSA against SSA, MFO, PSO, FA, and HHO, we plotted graphs of solution's quality (i.e., makespan) versus the number of iterations for the three datasets, as shown in Figures 2–16. From the convergence curves of the synthetic workload shown in Figures 2–6, HHOSA converges faster than other algorithms for 200, 400, 600, 800, and 1000 cloudlets. Besides, for NASA Ames iPSC/860, HHOSA converges at a faster rate than PSO, SSA, MFO, FA, and HHO for 500, 1000, 1500, 2000, and 2500 cloudlets, as depicted in Figures 7–11. Moreover, for the HPC2N real workload, HHOSA converges faster than other algorithms when the jobs vary from 500 to 2500, as shown in Figures 12–16. This indicates that the presented HHOSA generates better quality solutions and converges at a faster rate than other compared algorithms across all the workload instances.

To evaluate the algorithms, the performances of each algorithm were compared in terms of makespan. The values obtained for this performance metric are as reported in Tables 5–7. The results given in Tables 5–7 state that HHOSA usually can find better average makespan than other evaluated scheduling algorithms, namely, PSO, SSA, MFO, FA, and HHO. This means that the HHOSA takes less time to execute the submitted jobs and outperforms all the other scheduling algorithms in all the test cases. More specifically, the results demonstrate that the HHO is the second best. We also find that MFO performs a little better than SSA in most of the cases; both of them fall behind the HHO algorithm. Moreover, in almost all the test cases, FA is ranked far below SSA and PSO falls behind FA. This reveals that the average values of makespan using HHOSA are more competitive with those of the other evaluated scheduling methods.

The PIR(%) based on makespan of the HHOSA approach as it relates to the PSO, SSA, MFO, FA, and HHO algorithms is presented in Tables 8–10. For the synthetic workload, the results in Table 8 show that the HHOSA algorithm produces 92.51%–96.75%, 74.76%–85.15%, 66.10%–82.08%, 61.87%–76.81%, and 18.89%–23.87% makespan time improvements over the PSO, FA, SSA, MFO, and HHO algorithms, respectively. For the execution of NASA iPSC real workload (shown in Table 9), HHOSA shows 85.36%–93.24%, 66.99%–77.01%, 66.93%–74.69%, 65.31%–74.31%, and 15.05%–24.70% makespan time improvements over the PSO, FA, SSA, MFO, and HHO algorithms, respectively. In addition, the HHOSA algorithm gives 88.30%–94.09%, 79.83%–82.47%, 76.36%–80.83%, 75.44%–77.75%, and 13.55%–20.85% makespan time improvements over the PSO, FA, SSA, MFO, and HHO approaches for the HPC2N real workload shown in Table 10. That is to say, the performance of HHOSA is much better than that of the other methods.

### 5.4. Influence of the HHOSA Parameters

In this section, the performance of HHOSA is evaluated through changing the values of its parameters, where the value of population size is set to 50 and 150 while fixing the value of *β*=0.85. On the contrary, *β* is set to 0.35, 0.50, and 0.95 while fixing the population size to 100. The influence of changing the parameters of HHOSA using three instances (one from each dataset) is given in Table 11. From these results, we can notice the following: (1) By analyzing the influence of changing the value of swarm size Pop, it is seen the performance of HHOSA is improved when Pop is increased from 100 to 150, and this can be observed from the best, average, and worst values of makespan. In contrast, makespan for the swarm size equal to 50 becomes worse than that of swarm size equal to 100. (2) It can be found that when *β*=0.35, the performance of HHOSA is better than that when *β*=0.85 as shown from the best makespan value at HPC2N, as well as the best and worst makespan values at NASA iPSC. Also, in the case of *β*=0.5 and 0.95, the HHOSA provides better makespan values in four cases compared with the *β*=0.85 case as given in the table. From these results, it can be concluded that the performance of the proposed HHOSA at *β*=0.85 and Pop=100 is better than that at other values.

To summarize, the results herein obtained reveal that the developed HHOSA method can achieve near-optimal performance and outperforms the other scheduling algorithms. More precisely, it performs better in terms of minimizing the makespan while maximizing the utilization of resources. This allows us to infer that the hybrid HHOSA approach is an effective and efficient strategy for scheduling of jobs on IaaS cloud computing.

## 6. Conclusions

This paper proposes an alternative method for job scheduling in cloud computing. The proposed approach depends on improving the performance of the Harris hawks optimizer (HHO) using the simulated annealing algorithm. The proposed HHOSA algorithm has established its performance since it utilizes several operators which have high ability during the searching process to balance between the exploitation and the exploration. This leads to enhancing the convergence rate towards the optimal solution as well as the quality of the final output. Motivated from these improvements, this study proposes the HHOSA approach for addressing the problem of cloud job scheduling. To assess the performance of our method, a set of experimental series are performed using a wide range of instances ranging from 200 to 1000 cloudlets for synthetic workload and up to 2500 cloudlets in case of standard workload traces. Besides, it is validated over five well-known metaheuristics including MFO, SSA, FA, PSO, and the traditional HHO. The simulation results provide evidence about the high quality of the developed approach over all the other methods. According to the high performance obtained by the developed HHOSA algorithm, it can be extended in the future to handle other optimization issues in the cloud computing paradigm such as workflow scheduling and energy consumption. In addition, it is expected that HHOSA will be applied to other optimization problems in various research directions such as fog computing, Internet of things (IoT), feature selection, and image segmentation.

## Figures and Tables

**Algorithm 1:**
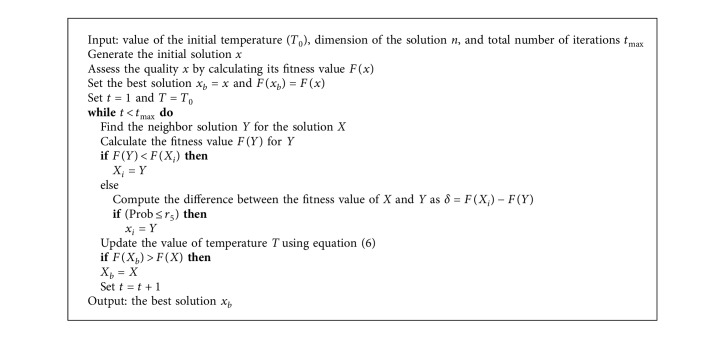
SA method.

**Algorithm 2:**
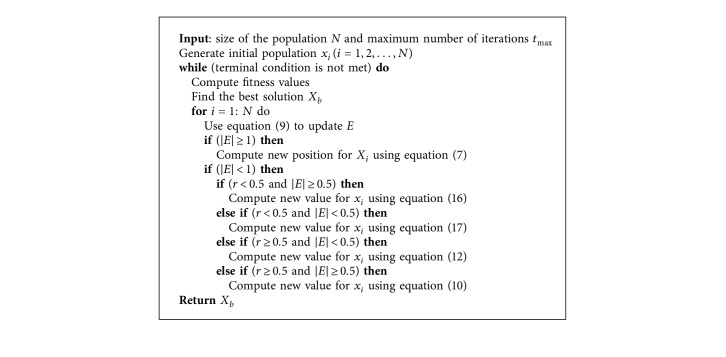
Steps of the HHO algorithm [17].

**Algorithm 3:**
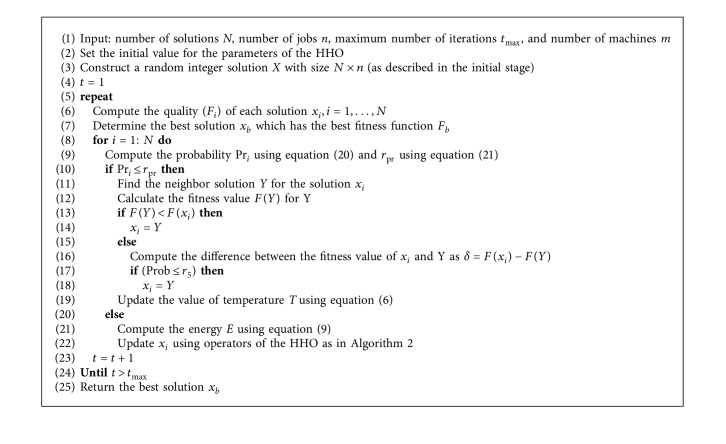
HHOSA scheduler for job scheduling in cloud computing.

**Table 1:** Experimental parameter settings.

Cloud entity	Parameters	Values
Data center	No. of data centers	1
	No. of hosts	2

Host	Storage	1 TB
	RAM	16 GB
	Bandwidth	10 Gb/s
	Policy type	Time shared

VM	No. of VMs	25
	MIPS	100 to 5000
	RAM	0.5 GB
	Bandwidth	1 Gb/s
	Size	10 GB
	VMM	Xen
	No. of CPUs	1
	Policy type	Time shared

**Table 2:** Synthetic workload settings.

Parameters	Values
No. of cloudlets (jobs)	200 to 1000
Length	1000 to 20000 MI
File size	300 to 600 MB

**Table 3:** Description of the real parallel workloads used in performance evaluations.

Log	Duration	CPUs	Jobs	Users	File
NASA iPSC	Oct 1993–Dec 1993	128	18,239	69	NASA-iPSC-1993-3.1-cln.swf

HPC2N	Jul 2002–Jan 2006	240	202,871	257	HPC2N-2002-2.2-cln.swf

**Table 4:** Parameter settings of each MH method evaluated.

Algorithm	Parameter	Value
PSO	Swarm size	100
	Cognitive coefficient *c*_1_	1.49
	Social coefficient *c*_2_	1.49

FA	Swarm size	100
	*α*	0.5
	*β*	0.2
	*γ*	1

SSA	Swarm size	100
	*c*_1_, *c*_2_, and *c*_3_	[0, 1]

MFO	Swarm size	100
	*b*	1
	*a*	−1 ⟶ 0–2

HHO	Swarm size	100
	*E*_0_	[−1, 1]

HHOSA	Swarm size	100
	*E*_0_	[−1, 1]
	*β*	0.85

**Table 5:** Best values of makespan for the HHOSA algorithm and compared algorithms.

	Instances	PSO	FA	SSA	MFO	HHO	HHOSA
Synthetic	200	59.64	56.49	47.86	48.39	38.10	33.12
	400	125.52	112.26	108.40	99.34	79.25	65.40
	600	190.63	176.91	162.81	158.01	113.51	97.74
	800	238.09	234.49	226.58	216.99	145.30	128.24
	1000	305.77	295.40	276.70	273.49	182.61	161.67

NASA iPSC	500	82.56	74.94	67.67	66.84	54.64	44.94
	1000	171.04	156.75	146.40	140.84	107.12	91.08
	1500	258.61	241.37	234.42	230.50	157.69	139.14
	2000	349.12	338.03	322.88	307.89	201.81	188.14
	2500	451.38	432.00	416.74	400.70	261.21	238.47

HPC2N	500	8946.52	8760.62	8001.93	8053.29	5572.43	4890.72
	1000	20793.74	19682.11	18496.02	18095.98	12253.81	10648.83
	1500	33361.63	31727.51	29824.93	29295.83	19732.12	17206.45
	2000	48135.05	45749.89	43808.80	42632.18	28076.55	25031.56
	2500	62713.88	60024.49	58845.85	58104.48	36228.46	32769.49

**Table 6:** Average values of makespan for the HHOSA algorithm and compared algorithms.

	Instances	PSO	FA	SSA	MFO	HHO	HHOSA
Synthetic	200	65.62	59.56	56.61	55.17	42.13	34.08
	400	133.78	122.41	121.51	115.72	84.22	67.99
	600	200.39	186.68	180.46	179.55	123.57	102.96
	800	261.08	247.77	242.60	236.93	161.93	135.62
	1000	325.96	311.34	306.18	297.32	199.92	168.16

NASA iPSC	500	90.68	78.36	78.33	77.57	58.51	46.93
	1000	181.61	165.28	163.53	163.90	117.04	95.04
	1500	275.67	257.90	253.43	253.96	177.10	145.70
	2000	369.50	352.45	346.74	342.04	234.68	199.35
	2500	470.77	446.78	442.03	433.85	291.10	253.03

HPC2N	500	9856.44	9247.51	8955.87	8909.51	6116.11	5078.28
	1000	21755.53	20489.89	19965.24	19800.62	13591.03	11245.99
	1500	34697.98	32882.46	32586.68	31774.13	21383.15	18020.64
	2000	49706.00	47499.34	46803.83	46919.56	30552.34	26397.05
	2500	65999.23	62867.26	62437.16	61628.58	39698.23	34960.06

**Table 7:** Worst values of makespan for the HHOSA algorithm and compared algorithms.

	Instances	PSO	FA	SSA	MFO	HHO	HHOSA
Synthetic	200	70.01	65.16	67.63	62.35	47.85	36.84
	400	139.31	131.25	131.74	137.68	91.24	74.56
	600	208.36	199.44	195.70	200.54	137.98	111.43
	800	270.97	258.75	261.55	261.78	176.48	147.32
	1000	343.91	320.15	332.00	334.82	228.45	177.86

NASA iPSC	500	96.88	87.83	89.68	87.57	64.94	49.09
	1000	194.88	175.43	178.62	187.98	137.02	103.91
	1500	290.92	274.93	266.68	290.62	199.25	161.69
	2000	389.26	365.76	370.27	372.62	276.77	209.73
	2500	497.98	469.99	475.52	470.00	337.07	270.55

HPC2N	500	10437.89	9729.10	9978.12	10015.48	6935.65	5338.96
	1000	22614.70	21187.87	21164.00	21352.51	14824.45	12335.51
	1500	35691.54	33880.20	34633.10	35055.64	23411.63	19072.55
	2000	51169.14	49156.43	49927.90	50376.81	35356.93	28300.92
	2500	68804.23	66036.35	65412.07	68422.54	44466.31	37691.71

**Table 8:** PIR(%) on makespan for synthetic workload.

	200	400	600	800	1000
PIR (%) over PSO	92.55	96.75	94.62	92.51	93.84
PIR (%) over FA	74.76	80.03	81.31	82.69	85.15
PIR (%) over SSA	66.10	78.71	75.27	78.88	82.08
PIR (%) over MFO	61.87	70.19	74.38	74.70	76.81
PIR (%) over HHO	23.62	23.87	20.01	19.40	18.89

**Table 9:** PIR(%) on makespan for real workload NASA iPSC.

	500	1000	1500	2000	2500
PIR (%) over PSO	93.24	91.08	89.20	85.36	86.05
PIR (%) over FA	66.99	73.90	77.01	76.81	76.57
PIR (%) over SSA	66.93	72.06	73.94	73.94	74.69
PIR (%) over MFO	65.31	72.45	74.31	71.58	71.46
PIR (%) over HHO	24.70	23.15	21.55	17.73	15.05

**Table 10:** PIR(%) on makespan real workload HPC2N.

	500	1000	1500	2000	2500
PIR (%) over PSO	94.09	93.45	92.55	88.30	88.78
PIR (%) over FA	82.10	82.20	82.47	79.94	79.83
PIR (%) over SSA	76.36	77.53	80.83	77.31	78.60
PIR (%) over MFO	75.44	76.07	76.32	77.75	76.28
PIR (%) over HHO	20.44	20.85	18.66	15.74	13.55

**Table 11:** Influence of the variant value of the parameters.

Instance	Makespan	*β*=0.85, Pop=100	Pop		*β*		
			50	150	0.35	0.55	0.95
HPC2N (500 jobs)	Best	4890.72	4945.263	4866.144	4881.952	4912.909	4946.269
	Average	5078.28	5200.724	5063.752	5094.164	5130.123	5162.682
	Worst	5338.96	5579.552	5317.88	5377.362	5350.381	5832.608

NASA iPSC (1000 jobs)	Best	91.08	91.16535	90.84299	90.40409	90.64667	90.79272
	Average	95.04	97.02455	94.96202	96.01011	96.03121	95.43977
	Worst	103.91	104.1675	101.4676	101.8874	101.7581	101.617

Synthetic (1000 jobs)	Best	161.67	162.9857	158.7836	162.6296	159.7758	161.2992
	Average	168.16	172.9459	164.9955	169.4086	167.829	168.612
	Worst	177.86	185.5133	173.191	184.2198	180.6842	177.6314

## Data Availability

The data used to support the findings of this study are available from the authors upon request.
